# Chronic vagus nerve stimulation in patients with heart failure: challenge or failed translation?

**DOI:** 10.3389/fcvm.2023.1052471

**Published:** 2023-07-18

**Authors:** Zhihong Wu, Jiaying Liao, Qiming Liu, Shenghua Zhou, Mingxian Chen

**Affiliations:** ^1^ Department of Cardiovascular, The Second Xiangya Hospital of Central South University, Changsha, China; ^2^ Department of Nephrology, The Second Xiangya Hospital of Central South University, Changsha, China

**Keywords:** vagus nerve stimulation, heart failure, anti-inflammatory, tragus nerve stimulation, translation

## Abstract

Autonomic imbalance between the sympathetic and parasympathetic nervous systems contributes to the progression of chronic heart failure (HF). Preclinical studies have demonstrated that various neuromodulation strategies may exert beneficial cardioprotective effects in preclinical models of HF. Based on these encouraging experimental data, vagus nerve stimulation (VNS) has been assessed in patients with HF with a reduced ejection fraction. Nevertheless, the main trials conducted thus far have yielded conflicting findings, questioning the clinical efficacy of VNS in this context. This review will therefore focus on the role of the autonomic nervous system in HF pathophysiology and VNS therapy, highlighting the potential reasons behind the discrepancy between preclinical and clinical studies.

## 1. Introduction

Heart failure (HF) represents a major public health problem associated with high morbidity, mortality and health care-related costs (1). It has been estimated that greater than 26 million patients suffer from HF worldwide. HF poses a substantial economic burden on patients and society. Expenditures associated with HF are thought to exceed $30 billion per year, and this value is expected to double in 2030 (2, 3). Despite important advances in medical and device-based therapies, hospitalizations and readmissions in patients living with HF continue to increase, particularly in patients aged ≥65 years (4, 5).

It is widely accepted that HF is characterized by an autonomic imbalance with a sustained increase in sympathetic drive and by withdrawal of parasympathetic activity (6). Decreased vagal tone is related to increased mortality in patients with HF (7). Given the evidence suggesting that increased vagal activity could reduce the risk of heart-related mortality, there is increasing interest in vagus nerve stimulation (VNS), which targets autonomic imbalance (6, 7). The use of VNS is supported by a strong rationale, consistent experimental data and encouraging preliminary clinical findings. However, a recent INOVATE-HF trial failed to demonstrate successful translation from animals to clinical studies (8). Therefore, whether device-based modulation of the VNS is a viable therapeutic strategy for patients with HF remains an important question. In this review, we will discuss the reasons for the potential reasons.

## 2. Autonomic dysfunction and heart failure

It has been considered for decades that HF is characterized by autonomic imbalance and hormonal hyperactivity. Autonomic dysfunction has been regarded as a manifestation of the clinical syndrome of HF, presumably as a consequence of hemodynamic changes associated with alterations in cardiac function (9, 10). Autonomic dysfunction is characterized by sympathetic hyperactivity and vagal withdrawn, whereas the hormonal response involves the activation of the renin-angiotensin-aldosterone system (RAAS) and sympathetic activation (11). The sympathetic nervous system innervates the adrenal glands and modulates the production of neurohormonal responses. Although the precise mechanisms involved in sympathovagal imbalance in HF patients remain to be clarified, consistent evidence suggests a key role played by the abnormal function of various reflex systems, including baroreflex, chemoreflex, and ergoreflex, as well as of their central integration, which may directly affect autonomic function (12–14).

At the cardiac level, cardiac ganglionated plexi (GPs) exist in the fat pads around the heart and constitute the so called “intrinsic cardiac nervous system”. GPs connect with the intrathoracic extracardiac ganglia (the sympathetic paravertebral ganglia). The nodose ganglia (the inferior ganglia of the vagus nerve) are extrathoracic and extracardiac while the dorsal root ganglia are intrathoracic and extracardiac. At each level, the system has the ability to modulate cardiac activity with efferent feedback loops. GPs coordinate the sympathetic and parasympathetic inputs received from the rest of the cardiac ANS (15).

Sympathetic hyperactivity produces cardiac toxicity, which induces interstitial fibrosis, cardiac apoptosis, and inflammation (16). On the other hand, as cardiac output becomes less efficiently induced by HF, a reduction in renal reperfusion increases the secretion of renin and activates the RAAS (17, 18). Activation of the RAAS may damage the myocardium. The potential mechanisms include the induction of chronic energy starvation, ventricular fibrosis, oxidative stress, and proinflammatory activity (19). Both overactivated sympathetic output and overproduction of RAAS aggravate the development of HF. Therefore, the effects of inhibition of sympathetic output and RAAS are potentially beneficial. Despite the pivotal role of drugs as a landmark therapy in HF patients, the residual risk for these patients remains high. Therefore, the role of other devices in modulating autonomic function should not be overlooked. Neuromodulation methods, including baroreflex activation therapy, left stellate ganglion block and renal sympathetic denervation, have been demonstrated to benefit chronic heart failure in experimental studies (20, 21).

Vagal withdrawal is also a critical element in the pathophysiology of chronic HF. Lower vagal activity is associated with unfavorable long-term prognostic implications for patients with HF (22, 23). Over the past several decades, great interest has emerged in modulating vagal activity as a therapeutic target for the treatment of HF. It has long been recognized that electrical VNS can prevent sudden cardiac death in conscious dogs and improve survival in rats with chronic HF (24, 25). Numerous potential sites of abnormal vagal control are noted, including the central nervous system, preganglionic fibers, postganglionic fibers and intracellular signaling pathways. Electrical stimulation of postganglionic fibers resulted in larger responses in the HF group compared to controls (26, 27). The potential benefit from enhanced vagal activity may involve the improvement of left ventricular dysfunction and structural remodeling.

## 3. Potential mechanisms of VNS in heart failure

Multiple mechanisms responsible for the protective effects of VNS on failing hearts have been observed (28). It has been accepted that VNS directly leads to improved parasympathetic tone and reflexes. VNS not only ameliorates autonomic dysfunction but also results in greater nitric oxide expression, improvement of RAAS and modulation of inflammatory cytokines (29–31). Moreover, recent studies have demonstrated that VNS can reduce apoptosis, inhibit oxidative stress, promote cardiac electrical stability and suppress stellate ganglion nerve activity (29). This finding indicated that vagal nerve stimulation might improve the outlook of patients with congestive heart failure (30).

### 3.1. VNS potentially inhibits sympathetic nervous activity

VNS could improve autonomic imbalance in failing hearts. The stimulation electrode is implanted in the mid-cervical portion of the vagal nerve and delivers a biphasic current that continuously cycles between on and off periods. Vagal afferent activation generated by VNS projects to the medulla located in the brainstem (31). The medulla contains cell bodies of the sympathetic and parasympathetic nervous systems. The nucleus tractus solitarius (NTS) of the medulla receives vagal afferent input and integrates the information. Neural connections from the NTS activate sympathetic neurons located in the rostral ventrolateral medulla (RVM) and inhibit parasympathetic neurons located in the dorsal vagal nucleus (DVN) and nucleus ambiguous (NA). VNS not only appears to increase the vagal tone to the heart but also may decrease sympathetic activity to some extent (32, 33).

The cervical vagal nerve contains both afferent and efferent fibers. Vagal fibers include A-, B- and C-fibers (34). Different electrical parameters activate different fibers. The cardiac response to cervical VNS presents a dynamic interaction between afferent mediated decreases in central parasympathetic drive and suppressive effects evoked by direct stimulation of parasympathetic efferent axons to the heart. The neural fulcrum is defined as the functional balance between afferent and efferent fibre activation. At low intensities and higher frequency VNS, HR increased during the VNS active phase owing to afferent modulation of parasympathetic central drive. As intensity increased further, HR was reduced during the active phase of VNS (35).

### 3.2. VNS modulates nitric oxide synthase expression

Nitric oxide (NO) plays a critical role in normal physiological functions and pathophysiological development in the heart. There are 3 distinct isoforms of nitric oxide synthase (NOS): neural NOS (nNOS), inducible NOS (iNOS), and endothelial NOS (eNOS) (36, 37). NO produced from eNOS contributes to regulating cell growth and apoptosis. Cardiomyocytes constitutively express eNOS, which enhances myocardial relaxation and modulates coronary perfusion (38). Endothelial NOS importantly regulated the development of HF. Both inflammatory cells and cardiac myocytes can express iNOS. A study has shown that iNOS overexpression in cardiomyocytes is related to ventricular fibrosis, left ventricular hypertrophy, chamber dilation and a cardiomyopathic phenotype (39). In dogs with heart failure induced by coronary microembolization, iNOS is obviously overexpressed, but eNOS is significantly downregulated (40). However, VNS in long-term therapy significantly improves the expression of eNOS and iNOS (40). nNOS is potentially upregulated in rats as well as in human failing hearts. A study demonstrated that preferential suppression of nNOS results in increased cardiac sensitivity to beta-adrenergic stimulation (41). nNOS was significantly overexpressed in the left ventricular myocardium after heart failure. However, VNS improved the abnormal expression of nNOS in the myocardium. Based on the above finding, HF could induce abnormal expression of three NOS isoforms. However, long-term VNS significantly tends to normalize the expression of NOS in the failing heart (40). nNOS can increase the release of Ach in parasympathetic neurons, while it could reduce the release of NE in sympathetic neurons. Interestingly, nNOS also may reverse impaired vagal and exaggerated sympathetic drive in the spontaneously hypertensive rat (42).

### 3.3. VNS suppresses activation of the renin-angiotensin system

As cardiac output becomes less efficient, a reduction in renal reperfusion increases the secretion of renin and activates the renin-angiotensin system (RAAS). Renin is a circulating aspartic proteinase that converts angiotensinogen to angiotensinogen I. Subsequently, angiotensinogen I is rapidly cleaved by angiotensin-converting enzyme to generate angiotensinogen II (Ang II). The effects of Ang II include vasoconstriction, ventricular remodeling, fibrosis, endothelin generation and sympathetic nervous action. Ang II contributes to enhancing sympathetic outflow in HF via central and peripheral effects. Mounting evidence demonstrates that AngII contributes to the increased SNA in CHF by acting in different brain regions, including the PVN, RVLM and area postrema. Ang II facilitates sympathetic neurotransmission at adrenergic nerve endings (18, 43). VNS inhibited RAAS activation. Vagal afferents from the cardiopulmonary region are reported to exert a tonic restraint on the release of renin. Vagal blockade significantly increased plasma renin activity in heart failure dogs (44, 45). VNS treatment decreased plasma Ang II levels in dog models. Therefore, inhibition of the renin-angiotensin system by VNS represents an additional therapeutic pathway (46).

### 3.4. VNS exerts an anti-inflammatory response

The vagal nerve facilitates the interactions of the neuroimmune system. It is now clear that VNS treats various inflammatory disorders of the organism. VNS contributes to controlling the inflammatory response by the cholinergic anti-inflammatory pathway through a vago-vagal reflex (47). Cholinergic receptors include muscarinic ACh (mACh) and nicotinic (nACh) receptors. mACh receptors are conventionally divided into five subtypes from M1 to M5 (48). However, the M2 and M3 receptor subtypes of the myocardium are important for cardiovascular diseases (49). nACh receptors have been identified in many cells. It is well known that α7nACh receptors of macrophages are involved in the cardioprotection conferred by VNS (50). ACh released from vagal terminals binds to the α7nACh receptors on macrophages and inhibits the production of inflammatory cytokines, including high-mobility group box 1 (HMGB1), TNF-α and interleukin-6 (IL-6) (51). Another anti-inflammatory pathway is the vagal-splenic pathway, which is a nonneuronal cholinergic pathway (52). In this pathway, VNS activates the splenic nerve, a sympathetic nerve issued from the celiac ganglion (53). Norepinephrine is released from the splenic nerve and binds to β2 receptors of T-lymphocytes of the spleen, resulting in the release of Ach. Ach binds to α7nAChR of macrophages to inhibit the release of TNF-α (54, 55).

## 4. Preclinical studies

Preclinical studies suggested that chronic VNS could exert protective effects on the heart in animal models of heart failure (see Table 1). In 2004, an experimental study reported by Li et al. showed that chronic VNS resulted in significant improvement in cardiac function and decreased mortality in a rat model of CHF after large myocardial infarction (25). Zhang et al. investigated the effect of chronic VNS in a canine rapid ventricular pacing model of heart failure. Chronic VNS significantly improved left ventricular (LV) ejection fraction and reduced LV end-diastolic and end-systolic volumes (46). VNS markedly attenuated the increased levels of plasma catecholamine, angiotensin II and C-reactive protein. Sabbah and colleagues established a canine model of HF produced by multiple sequential coronary microembolizations (56). After 3 months of chronic VNS therapy, LV end-systolic volume decreased, and LV ejection fraction increased. Several biomarkers of heart failure were positively attenuated by VNS.

**Table 1:** Characteristics of the preclinical studies, clinical experiences and clinical trials in the treatment of HF by VNS.

Authors	Study design	Subjects	VNS number	Diagnosis	Assessment	Intervention	Time of Intervention	Main outcomes	Summary
Li et al. (25)	Controlled experimental study	Rats	11	HF induced by myocardial infarction	Cardiac remodeling and long-term survival	Right-sided VNS	6 weeks	VNS improved the long-term survival and cardiac remodeling.	VNS is a feasible method in rats study.
Zhang et al. (46)	Control experimental study	Dogs	8	HF induced by high-rate pacing	High-rate pacing/HF development	Right-sided VNS	8 weeks	VNS prevented HF development, improved autonomic control and reduced inflammatory effects.	VNS is a feasible and effective method in canine study.
Hamann et al. (57)	Controlled experimental study	Dogs	7	HF induced by microembolizations induced	LV structure and function	Right-sided VNS	6 months	VNS improved LV structure and function, and biomarkers.	VNS is a feasible and effective method in canine study.
Schwartz et al. (59)	Single central, pilot study	Humans	8	HF	Feasibility, safety and efficacy of chronic VS in HF patients.	Right-sided VNS	6 months	Improvements in NYHA class, quality of life, LV structure.	VNS is a feasible and effective method in patients with HF.
De Ferrari et al. (60)	Multiple-centre, open-label, two-staged study	Humans	32	HF	Feasibility, safety and efficacy of chronic VS in HF patients.	Right-sided VNS	6 months	Improvements in NYHA class, quality of life, LVEF and LV volume. The improvements maintained for 1 year.	VNS in CHF patients is a safe, tolerable and effective method.
Premchand et al. (63)	Randomized and controlled trial	Humans	60	HF HF patients.	Safety and efficacy of left or right VNS in HF patients.	Left-sided or right-sided VNS	10 weeks	Improvements in HRV, 6 min walk distance and LVEF.	Both left- or right-sided VNS is feasible, tolerated, and effective.
Zannad et al. (66)	A Phase II, randomized clinical trial	Humans	96	HF	The primary endpoint with LVESD, and the second endpoint with exercise capacity, quality of life, 24-holter, and circulating biomarkers.	Right-sided VNS	6 months	No significance among echocardiographic parameters, but a significant improvement in quality of life.	It failed to demonstrate a significant effect on primary and secondary endpoint measures, but quality-of-life measures showed significant improvement.
Gold et al. (8)	A multinational, randomized trial	Humans	707	HF	Safety and efficacy of VNS among patients with HF	Right-sided VNS	18 months	Quality life and NYHA were improved, but LVESVI were not significant.	VNS does not reduce the rate of death or HF events in chronic HF patients.

CHF, chronic heart failure; HF, heart failure; HRV, heart rate variability; LV, left ventricular; LVEF, left ventricular ejection fraction; LVESD, left ventricular end systolic diameter; LVESVI, left ventricular end-systolic volume index; NYHA, New York Heart Association; VNS, vagus nerve stimulation.

Hamann et al. developed a canine model of HF induced by intracoronary microembolizations that has been used for chronic vagal nerve stimulation without causing heart rate reduction. They found that VNS treatment significantly increased LV ejection fraction, reduced left ventricular chamber dimension, and improved biomarkers of inflammatory cytokines and cellular apoptosis in heart failure (57). These preclinical studies indicated that VNS is an effective and feasible treatment for chronic heart failure. VNS could induce bradycardia in chronic HF. Therefore, β blockers may potentially cover the beneficial effects of VNS on chronic HF. Sugimachi et al. performed a study and found that VNS exerted additional beneficial effects on HF with the β blockers treatment. They found that VNS achieved beneficial effects on the failure heart independently of its anti-beta-adrenergic mechanism (58).

## 5. Clinical experiences

VNS was approved for the treatment of patients with drug-refractory epilepsy in 1997 and medically refractory depression in 2005. Based on the efficacy of preclinical studies and the safety of VNS management in patients, clinical studies of the treatment of VNS were started (see Table 1).

Schwartz et al. reported a single-center pilot study of 8 patients with severe HF who were implanted with the Cardiofit system. This first-in-man experience of chronic VNS in patients with HF demonstrated that VNS significantly reduced the NYHA classification, markedly improved the quality of life and decreased left ventricular end-systolic volume (59). This result suggested that VNS treatment was feasible and appeared safe and tolerable. Given the beneficial results, De Ferrari et al. subsequently expanded the study and enrolled 32 patients with symptomatic HF and reduced LV ejection from multiple centers. At the preliminary 6-month follow-up, VNS significantly improved LV ejection fraction, LV end-systolic volume and 6 min walk test results, and these effects were maintained to the 1-year follow-up (60). These encouraging results suggested that VNS has clinical merit in HF treatment.

## 6. Clinical trials

Currently, most neurostimulation devices provide stimulation in an open-loop manner; however, closed-loop neurostimulation devices (i.e., modulate therapy in response to physiological changes) may provide more effective and efficient therapy. Herein, we focused on clinical trials of implantable closed-loop vagus nerve stimulation for the treatment of chronic heart failure (61, 62). The positive results of CARDIOFIT™ have led to further clinical trials, including ANTHEM-HF (Autonomic Neural Regulation Therapy of Enhance Myocardial Function in Heart Failure) (63–65), NECTAR-HF (Neural Cardiac Therapy for Heart Failure) (66, 67), and INOVATE-HF (Increase of Vagal Tone in Congestive Heart Failure) (34, 68) trials (see Table 1).

The ANTHEM-HF study was a prospective and open-label study enrolling 60 NYHA class II-III patients with an LV ejection fraction <40% and a QRS <130 ms (64). Patients followed for over 6 months were randomized to either left or right cervical VNS; no control group was included in this study. The stimulation protocol with an amplitude of 2.0 ± 0.6 mA at 10 Hz stimulation and with a duty cycle of 17.5% (14 s on and 66 s off) was used in the VNS system. LV ejection fraction significantly increased by 4.5% (*p* < 0.05), but no significant decrease in LV end-systolic volume was noted. Improvements in NYHA classification (77% of patients) and the Minnesota Living with Heart failure score were observed. Interestingly, no statistical significance was noted between left-sided and right-sided vagal stimulation. Owing to insufficient dosing of autonomic regulation therapy (ART) such as VNS, larger clinical studies need further to be studied. Recently, the ANTHEM-HFrEF study was designed to explore whether VNS using appropriate ART could improve morbidity and mortality as well as symptoms and function for patients with advanced HF. The ANTHEM-HFrEF study, with adaptive sample size selection, is an adaptive, open-label, randomized, controlled study (69).

The NECTAR-HF study was a prospective and double-blinded study enrolling 96 patients in NYHA class II-III, with an LV ejection fraction ≤35% and LV end-diastolic diameter >55 mm. All patients were implanted with a VNS device without the use of a right ventricular sensing lead and then randomly divided 2:1 into the active group and sham group for the first 6 months. For the second 6 months, all patients received VNS treatment. The stimulation parameters had an average amplitude of 1.42 ± 0.8 mA at 20 Hz and a duty cycle of 17% (10 s on and 50 s off). No significant change in the LV end-systolic dimension of the primary endpoint was noted. No significant differences in LV end-systolic and diastolic volume, LVEF or plasma biomarkers were noted as secondary endpoints, whereas significant improvements in NYHA functional class and quality of life were observed (67).

The INOVATE-HF study was a pivotal phase III multicenter study enrolling 707 patients with NYHA class III, LV ejection fraction <40% and LV end-diastolic diameter 50–80 mm. Patients were randomized 3:2 to either the VNS group or the sham implantation group. The primary endpoints of this study focused on complications at 90 days, all-cause mortality and HF hospitalizations at 12 months. This trial was stopped in the last year by the Steering Committee due to the results. There was no significant difference in primary efficacy between the VNS group and the control group. No significant difference in LV end-systolic volume index was observed between groups. However, significant improvements in NYHA classification, quality of life and 6 min walking distance were noted (8). VNS delivery was open-loop in ANTHEM-HF and NECTAR-HF, but VNS delivery was closed loop in INOVATE-HF. Open-loop delivery targeted at both central and peripheral nervous activity. Closed-loop delivery preferentially aimed at peripheral neural targets. It required a right ventricular intracardiac lead in order to synchronize VNS delivery to R-wave sensing.

## 7. Cervical VNS in patients with HF: A failed translation?

The results between preclinical studies and clinical trials are inconsistent. Moreover, the INOVATE-HF trial presented somewhat negative study results. Do the results mean that it fails to demonstrate a successful translation of VNS treatment? To better understand these results, there are some concerns that should not be ignored.

### 7.1. Dose issue

The dose‒response curve is estimated to determine the proper dosage and achieve the greatest possible benefit in pharmacological trials. A dose-response curve should also be generated for VNS treatment. However, given the different parameters of combinations, the “dose” of electrical therapies is considerably more complex than that noted for pharmacological therapies. The cervical vagal nerve contains both afferent and efferent fibers composed of A-, B- and C-fibers (34). Given that the threshold for stimulation varies inversely with fiber diameter, VNS at low-intensity stimulus initially activates A-fibers and gradually recruits B-fibers with higher intensity. As the intensity continues to increase, C-fibers are recruited (35). Owing to intrinsic properties and larger diameters, afferent fibers are preferentially activated at low stimulation thresholds, which subsequently increases vagal activity and suppresses sympathetic activity via CNS modulation (70). Regarding the frequency of stimulation, low frequencies (5–10 Hz) activate vagal afferents, whereas high frequencies (10–30 Hz) activate both vagal afferents and efferents (29, 30, 47, 71). In order to the demonstrate the effect of chronic VNS on central–peripheral neural network interactions for integrated control of the heart, Ardell et al. firstly proposed “neural fulcrum”. Based on frequency–amplitude–pulse width, the “neural fulcrum” is defined as the operating point, where a null heart rate response is reproducibly evoked during the on-phase of VNS. The fulcrum point stably maintains over the average 14 months of chronic VNS (72).

The stimulating lead and strength in NECTAR-HF and ANTHEM-HF studies were designed to stimulate afferent fibers, whereas lead and strength used in CardioFit and INOVATE-HF studies were designed to stimulate efferent fibers. In theory, afferent fiber stimulation would be more beneficial for decreasing sympathetic activity than efferent fiber stimulation. However, a large dose may damage vagal fibers. A stimulation frequency of 1–2 Hz was applied in the CardioFit system, and a stimulation current of 4.1 ± 1.2 mA was achieved at the end of titration. A stimulation frequency of 10 Hz was applied in ANTHEM-HF, and a current output of 2.0 ± 0.6 mA was achieved at the end of titration. NECTAR-HF used a frequency of 20 Hz and reached 1.2 ± 0.7 mA (see Table 2). The low intensity of stimulation applied in NECTAR-HF was related to B fibers. B fibers contributed to a lower heart rate and anti-remodeling effects. Although the low stimulation current in NECTAR was previously considered a cause for the negative findings, it was questioned by the neutral result of the INOVATE-HF study with a high stimulation amplitude (3.9 ± 0.7 mA). Therefore, other factors are responsible for the failure to achieve the different results of VNS in the HF long term (67, 73). Moreover, the duty cycle designed in the CardioFit study led to a reduced heart rate, whereas no reduction in heart rate was noted in the INOVATE-HF trial. Therefore, the results of chronic VNS in patients with HF are related to multiple factors, including stimulating parameters (current intensity, frequency, duty cycle), electrode design and stimulated-side selection. Research on the optimal dose of VNS needs further study. Despite the intended design, the stimulation in all 4 studies resulted in both afferent and efferent stimulation. Furthermore, the relative benefit of afferent vs. efferent stimulation (or both) has not been demonstrated clinically.

**Table 2:** Stimulation parameters of VNS in three clinical trials.

Parameters	ANTHEM-HF pilot study	NECTAR-HF	INOVATE-HF
Neural target	Central/peripheral	Central/peripheral	Peripheral
Delivery site	Left- or right-sided VNS	Right-sided VNS	Right-sided VNS
**Delivery intensity**			
Amplitude (milliamperes)	2.0 ± 0.6	1.4 ± 0.8	3.9 ± 1.0
Frequency (Hz)	10	20	From 1 to 2
Duration (ms)	250	300	500
Duty cycle	17.5%	17%	25%
On-time/off time (s)	18/62	10/50	Variable
Electrode polarity	Caudal	Caudal	Cephalad
Model of delivery	Open loop/cyclic	Open loop/intermittent	Closed loop/intermittent

HF, heart failure; VNS, vagus nerve stimulation.

### 7.2. Patient selection

Optimal patient selection also holds the key to obtaining better outcomes for patients with HF. The benefit of patients with HF from VNS is likely to be associated with the extent of neuro-hormonal derangement. The patients with low levels of autonomic imbalance may not benefit from VNS therapy. VNS is known to relieve the inflammatory response. Patients with evidence of cardiac inflammation may benefit much more (74). Furthermore, patients with long-standing heart failure may be refractory to all therapies, including VNS treatment (75). Finally, lifestyle changes, such as exercise, can increase vagal activity and reduce mortality in patients with HF. Whether VNS combined with exercise will offer benefits remains an open question (76).

## 8. More research for possible improvement

Resting heart rate (HR) is a simple index reflecting the external autonomic regulation of the intrinsic heart rate at the sinus node level that is achieved with the combined activity of the sympathetic nervous system (SNS) and the parasympathetic nervous system (PNS). In fact, heart rate has two components -the intrinsic and the extrinsic component. Intrinsic HR is the HR measured in the absence of sympathetic and parasympathetic inputs (achieved by denervation or pharmacologic blockade). In healthy human subjects, this is approximately 100 bpm and is age- and gender-dependent (77). Intrinsic HR depends on sinus node automaticity and on the ionic transportations through the cell membrane that continuously generate the action potential. Denervated transplanted hearts, which lack the SNS and PNS function, beat fast at 100 bpm and their frequency exclusively depends on intrinsic automaticity. It is the impact of PNS tone during rest that dominates SNS and sinus node automaticity and decreases this frequency to 50–60 bpm. The slope of action potential depolarization is determined from If channels. SNS and PNS increase or decrease the heart rate by changing this slope. The influences of the SNS and PNS on HR have been proposed to be defined by the following formula: HR = *m* × *n* × HR0; where *m* is the sympathetic influence (>1), *n* is the parasympathetic influence (<1), and HR0 is the intrinsic HR (78). Given the heart rate physiology analyzed above, it is rational to propose that the VNS effectiveness on the autonomic nervous system's status is reflected by the resting HR. In other words, VNS may be therapeutic and efficient if a critical decrease of the post-VNS baseline resting HR is achieved. In this case, SNS and PNS may reach a new (and therapeutic) balance. The ANTHEM-HF study reported an improvement in 24 h HR from 78 bpm to 70 bpm (*p *=* *<0.0005) after 12 months of VNS, while SDNN from HRV increased from 95 ms to 109 ms (*p* = <0.01). This information is not presented in the INOVATE-HF study, so it is not possible to determine the effectiveness of PNS stimulation that was applied in this study.

It is currently unknown what the most appropriate stimulation protocol would be. Indeed, all studies applied protocols with variances in amplitude, frequency of stimulation, and afferent—efferent vagus activity targeting (Schwartz, ANTHEM-HF, NECTAR-HF, INOVATE-HF). The effectiveness of the applied protocol may be estimated according to the above comment 1 from ΔHR while ΔHR = HR baseline—HR post VNS. Additionally, measuring the differences in plasma concentrations of catecholamines pre- and post-VNS (Δ Nor-Epinephrine and Δ Epinephrine) may also serve well as a useful biomarker of a therapeutic efficient new SNS-VNS status, especially if these catecholamines are found to decrease after VNS application. Before any VNS study's results be interpreted, it is necessary to clarify whether the applied VNS protocol was optimal and therapeutic. One simple way to quantify the response of the ANS to the applied VNS is by comparing the achieved differences in heart rate and catecholamines.

It is unclear whether the afferent or the efferent vagus nerve fibers targeting neurostimulation is cardio-protective. Afferent targeting VNS may be necessary for ANS central reset. Further research is required. Vagal function may include two distinct components: reflex vagal activity (79) and tonic vagal activity (80). The extent to which these activities are improved by VNS is unknown. Furthermore, it is unspecified which one contributes more to protection against mortality in HF patients. For example, reflex vagal activity may be protective during ischemia-induced arrhythmias, while tonic vagal activity may improve left ventricular properties, function, and dimensions. Heart Rate Turbulence may quantify reflex vagal function, while Deceleration Capacity of Heart Rate and RMSSD from HRV may quantify tonic vagal activity. Future VNS studies may include such Holter indices to investigate the improvement of tonic and reflex vagal activity after VNS. Therefore, more research need to be further studied to explore the really effectiveness of VNS in patients with heart failure.

## 9. Evolving strategy

**Figure 1:**
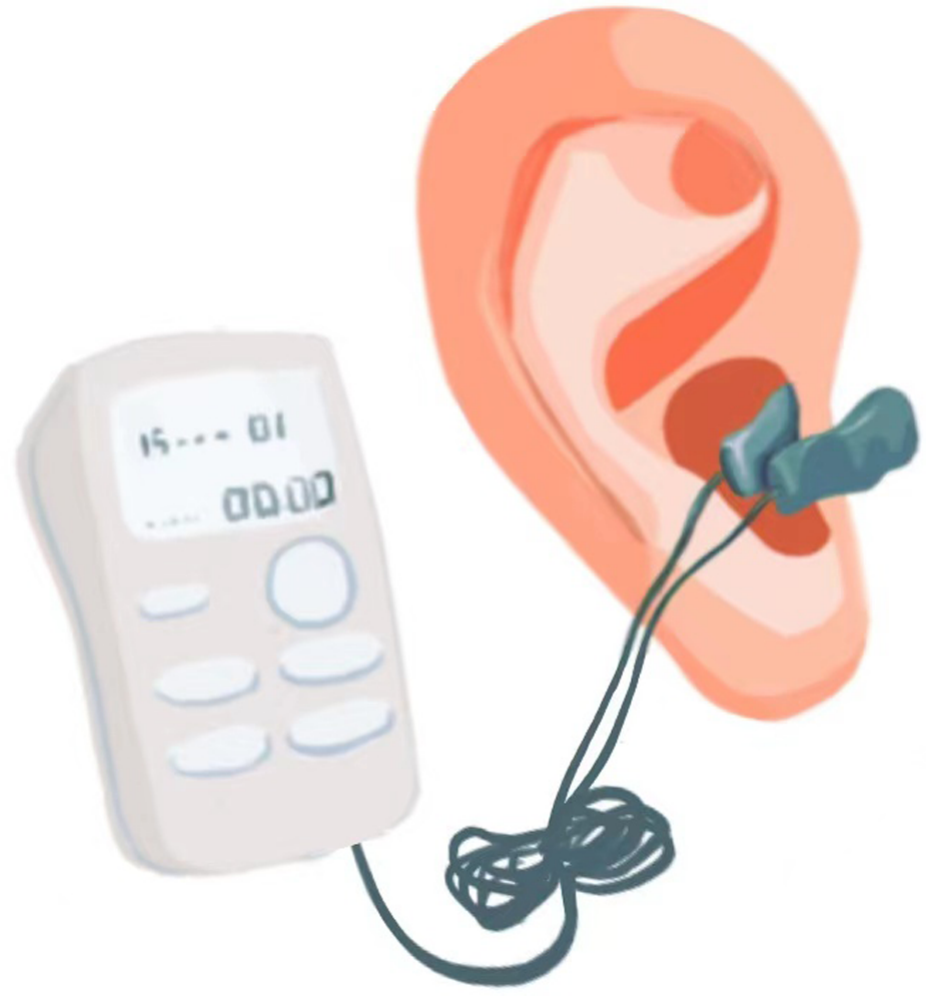
Tragus nerve stimulation at auricular branch of the vagus nerve.

**Figure 2:**
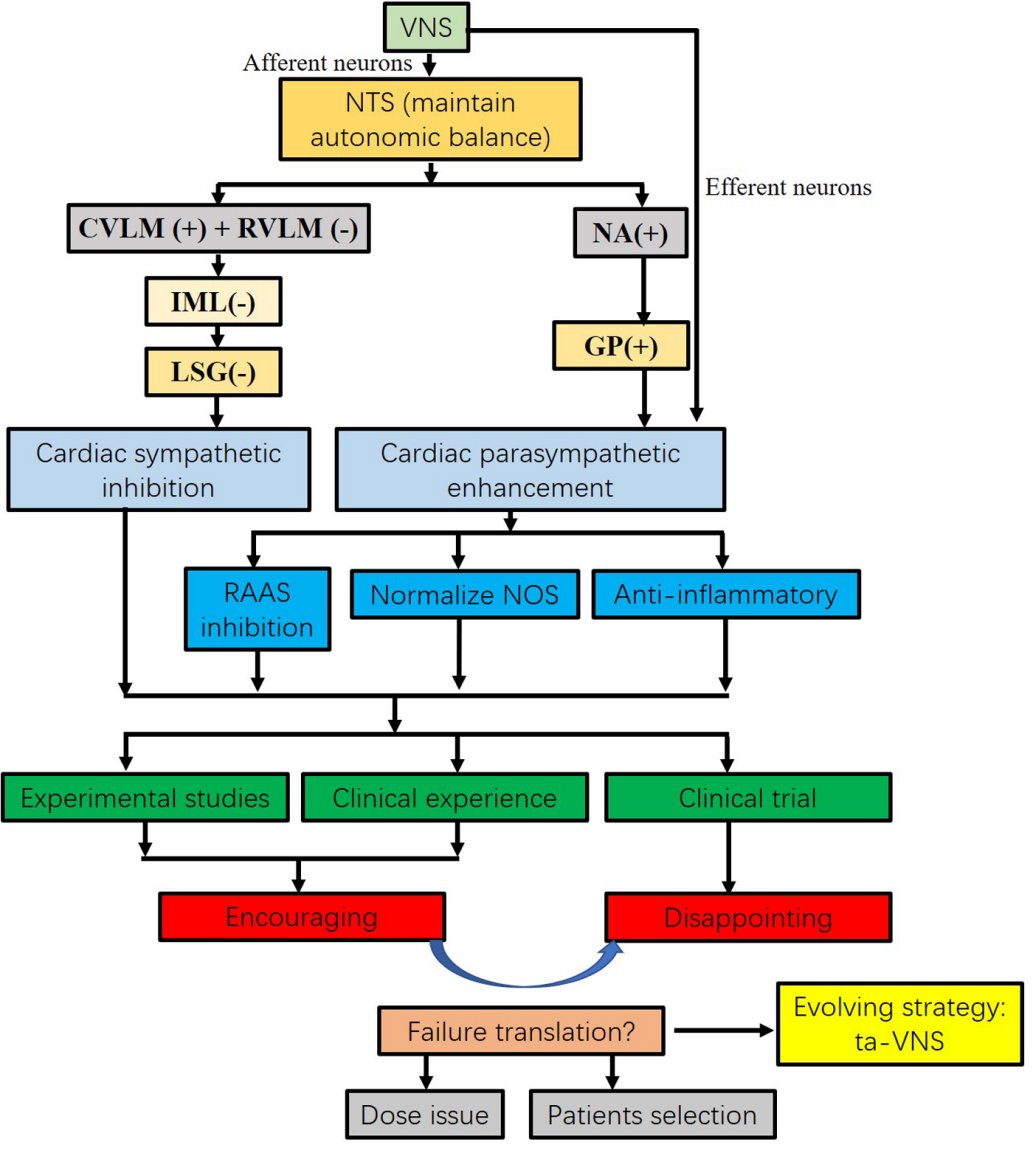
Flow chart of VNS on heart failure from concept to translation. VNS maintains autonomic balance. VNS significantly inhibits sympathetic nervous activity and enhances vagal tone. Increased vagal activity attenuates heart failure by RAAS inhibition, NOS normalization, and anti-inflammatory response. The results from the experimental studies and clinical experiences are encouraging. However, the results from recent clinical trials did not achieve the same benefit as experimental studies. Several reasons may contribute to the translation, including dose issues and patient selection. Instead of electrical VNS, tragus nerve stimulation is an evolving strategy. CVLM, caudal ventrolateral medulla; GP, ganglion plexus; IML, intermediolateral cell column; LSG, left stellate ganglion; Ta-VNS opens an era in the treatment of heart failure. NOS, nitric oxide synthase; NTS, nucleus of the solitary tract; RAAS, renin-angiotensin-aldosterone system; RVLM, rostroventrolateral medulla; ta-VNS, tragus nerve stimulation; VNS, vagus nerve stimulation.

The auricular branch of the vagus nerve (ABVN) on the outer ear is the only peripheral branch of the vagus nerve distributed on the skin (81). Transcutaneous VNS, a novel noninvasive neuromodulation (Figure 1), targets ABVN at the outer ear instead of resorting to VNS with surgery and impacts autonomic tone (82). We previously showed that low-level ta-VNS (LL-TS) suppressed AF by prolonging atrial effective refractory periods and reducing AF inducibility in a canine AF model induced by rapid atrial pacing (83). Po and his team demonstrated that LL-TS could effectively suppress atrial fibrillation and decrease inflammatory cytokines in patients with paroxysmal atrial fibrillation (84, 85). A recent clinical study by Jiang et al. demonstrated that LL-TS could reduce myocardial ischemia‒reperfusion injury in patients with ST-segment elevation myocardial infarction (86). This result indicated that LL-TS begins to gradually result in clinical efficacy. More importantly, the results of LL-TS in the treatment of cardiovascular diseases are encouraging. Beyond the protective effects in atrial fibrillation and acute myocardial infarction, LL-TS has also been applied to research left ventricular remodeling. Wang et al. showed that chronic intermittent LL-TS could attenuate left ventricular remodeling in conscious dogs with healed myocardial infarction (87). In a preclinical model of postinfarction cardiomyopathy, tragus stimulation was associated with attenuation of ANS imbalance (plasma NE) and neurohormonal activation (NT-proBNP) as well as improvement in LV function. However, the translational effects of LL-TS in the treatment of heart failure still require further study. More recently, LL-TS has emerged as an intriguing option in patients with chronic HF. LL-TS resulted in a significant improvement in global longitudinal strain, inflammatory cytokines, and quality of life in patients with heart failure with preserved ejection fraction (88, 89). The ta-VNS opens an era in the treatment of HF (Figure 2).

The different translation results of VNS treatment on HF, another reason was that the patients were not able to tolerate higher-intensity stimulation in clinical trials. As a result, VNS could not achieve the effective stimulation. In order to reduce the adverse effects and increase the tolerance of VNS, recently, selective VNS was also considered as a promising strategy (90–93). Selective VNS targets specific fiber to cause functionally specific effects. Selective VNS not only can reduce side effects, but also increase efficacy to some extent. Several methods, including spatially selective, fiber-selective, anodal block, neural titration, kilohertz electrical stimulation block, stimulation pulse parameters setting, and electrode array geometries changes, have been applied in the field of selective VNS. The development of selective VNS techniques will likely benefit patients in future, however, selective VNS is also a small research area. More and more studies should be performed its safety and efficacy in the treatment of cardiovascular diseases.

## 10. Conclusion

Experimental and clinical pilot studies of VNS yielded encouraging results in the treatment of HF. However, the results of large randomized clinical trials have been disappointing. The discrepancy between experimental and large clinical trials may be associated with optimal dosing of stimulation, appropriate patient selection, and study design. The clinical translation of VNS in the treatment of HF is a challenge. The era of vagus nerve stimulation for the treatment of heart failure is approaching; however, significant experimental and clinical research is still needed (69).

## Data Availability

The original contributions presented in the study are included in the article, further inquiries can be directed to the corresponding author.
